# Intertwined density waves in a metallic nickelate

**DOI:** 10.1038/s41467-020-19836-0

**Published:** 2020-11-26

**Authors:** Junjie Zhang, D. Phelan, A. S. Botana, Yu-Sheng Chen, Hong Zheng, M. Krogstad, Suyin Grass Wang, Yiming Qiu, J. A. Rodriguez-Rivera, R. Osborn, S. Rosenkranz, M. R. Norman, J. F. Mitchell

**Affiliations:** 1 Materials Science Division, Argonne National Laboratory, Lemont, IL 60439 United States; 2 Institute of Crystal Materials, State Key Laboratory of Crystal Materials, Shandong University, 250100 Jinan, Shandong China; 3 Department of Physics, Arizona State University, Tempe, AZ 85287 United States; 4 ChemMatCARS, The University of Chicago, Lemont, IL 60439 United States; 5 NIST Center for Neutron Research, National Institute of Standards and Technology, Gaithersburg, MD 20899 United States; 6 Department of Materials Science, University of Maryland, College Park, MD 20742 United States

**Keywords:** Electronic properties and materials, Magnetic properties and materials, Structure of solids and liquids

## Abstract

Nickelates are a rich class of materials, ranging from insulating magnets to superconductors. But for stoichiometric materials, insulating behavior is the norm, as for most late transition metal oxides. Notable exceptions are the 3D perovskite LaNiO_3_, an unconventional paramagnetic metal, and the layered Ruddlesden-Popper phases R_4_Ni_3_O_10_, (R = La, Pr, Nd). The latter are particularly intriguing because they exhibit an unusual metal-to-metal transition. Here, we demonstrate that this transition results from an incommensurate density wave with both charge and magnetic character that lies closer in its behavior to the metallic density wave seen in chromium metal than the insulating stripes typically found in single-layer nickelates like La_2-*x*_Sr_*x*_NiO_4_. We identify these intertwined density waves as being Fermi surface-driven, revealing a novel ordering mechanism in this nickelate that reflects a coupling among charge, spin, and lattice degrees of freedom that differs not only from the single-layer materials, but from the 3D perovskites as well.

## Abstract (web summary)

Layered Ruddlesden-Popper structure nickelates *R*_4_Ni_3_O_10_ (*R* = La,Pr) show an unusual metal-to-metal transition, but its origin has remained elusive for more than two decades. Here, the authors show that this transition results from intertwined density waves that arise from a coupling between charge and spin degrees of freedom

## Introduction

The central challenge in harnessing the unrivaled diversity of transition metal oxides (TMO) is understanding (and manipulating) how charge and spin influence their properties. Gaining such understanding and control is made difficult by the many highly correlated degrees of freedom ubiquitously present in TMO. As a case in point, the family of rare earth nickel oxide perovskites, RNiO_3_ (R = La, Pr–Lu), presents an important archetype TMO for investigating the nexus between charge localization and itineracy and their correlation with magnetism. These well-studied Ni^3+^ (d^7^) perovskites exhibit a commensurate antiferromagnetic insulating ground state for all R except La, and the ordering wavevectors (**q**_MIT_) driving the metal-insulator transition (MIT) and magnetic ordering are commensurate with respect to the parent cubic perovskite^1^. On the other hand, LaNiO_3_ is a good metal that remains rhombohedral at all temperatures. It shows no intrinsic, long-range ordered antiferromagnetism in bulk samples^2–5^, although evidence of short-range bond disproportionation associated with **q**_MIT_ has been reported^6^. By reducing the dimensionality, insulating ground states with commensurate stripe or checkerboard charge and spin-ordering are found in Sr-doped La_2_NiO_4_ (LSNO), the *n* = 1 member the quasi-2D Ruddlesden-Popper (R-P) series La_*n*+1_Ni_*n*_O_3*n*+1_, throughout its doping range between Ni^2+^(d^8^) and Ni^3+^ (d^7^)^7^. Incommensurate charge and spin modulations observed in LSNO have been shown to arise from microscopic mixtures of commensurate stripe phases^8^. Commensurate charge-stripes and spin-stripes have also been identified recently in another low-dimensional insulating nickelate, La_4_Ni_3_O_8_, also at 1/3-hole doping, albeit here Ni^1.33+^ (d^8.67^)^9,10^. Collectively through these systems, our current understanding of the rare earth nickelates has been framed as lying poised between localized and itinerant behavior and between magnetic order and disorder with both sectors governed by real-space charge and spin interactions.

Here, we report a dramatically different behavior in a higher order (*n* = 3) R–P nickelate, R_4_Ni_3_O_10_ (R = La, Pr, Nd), in which we find intertwined charge-order and spin-order developing at a metal-to-metal transition (MMT) whose precise mechanism has remained an open question for nearly 25 years^11–18^. Combining single crystal synchrotron x-ray and neutron diffraction, we find charge and spin-superlattice (SL) reflections below the MMT with incommensurate propagation vectors **q**_c_ = (0,*q*_*c*_,0) and **q**_s_ = (0,1–*q*_*s*_,0), respectively, with *q*_*c*_ = 2*q*_*s*_ as expected for a system with coupled charge and spin order. We present models for these intertwined density waves that reproduce the experimental observations semi-quantitatively. In the charge sector, our data are uniquely consistent with a CDW centered on the Ni sites, establishing that the low energy electronic degrees of freedom in this system lie on the transition metal rather than on oxygen. The lack of local moments on La unequivocally establishes that the SDW is associated with the Ni ions. A measured compositional dependence of the incommensurate **q**_c_ combined with DFT calculations argue that these itinerant systems order via a Fermi surface nesting mechanism, in this way resembling elemental chromium^19^ more than nickel oxides. As such, the unexpected behavior found in R_4_Ni_3_O_10_ represents an important bridge between the paramagnetism and latent charge-ordering and spin-ordering of 3D metallic LaNiO_3_ at higher nickel valence and the polaronic behavior found in quasi-2D R_2-*x*_Sr_*x*_NiO_4_ at lower nickel valence.

## Results

### Physical properties

**Fig. 1:**
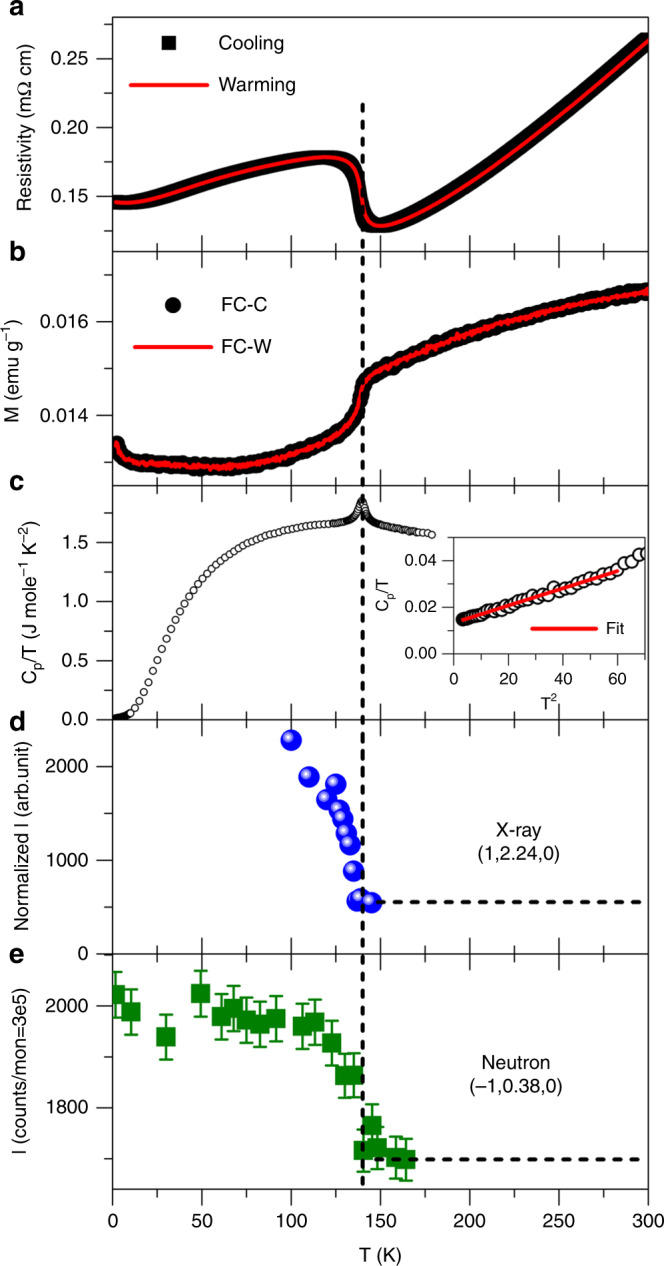
**Physical properties and order parameter of La**_**4**_**Ni**_**3**_**O**_**10**_. ab-plane (**a**), resistivity in zero field and (**b**), magnetic susceptibility measured under 0.4 T upon cooling and warming. **c** Heat capacity. Inset shows fit to low temperature data. **d** Temperature dependence of integrated intensity at (1, 2.24, 0) measured with x-rays. **e** Temperature dependence of integrated intensity at (−1, 0.38, 0) measured with neutrons. Error bars represent one standard deviation.

**Fig. 2:**
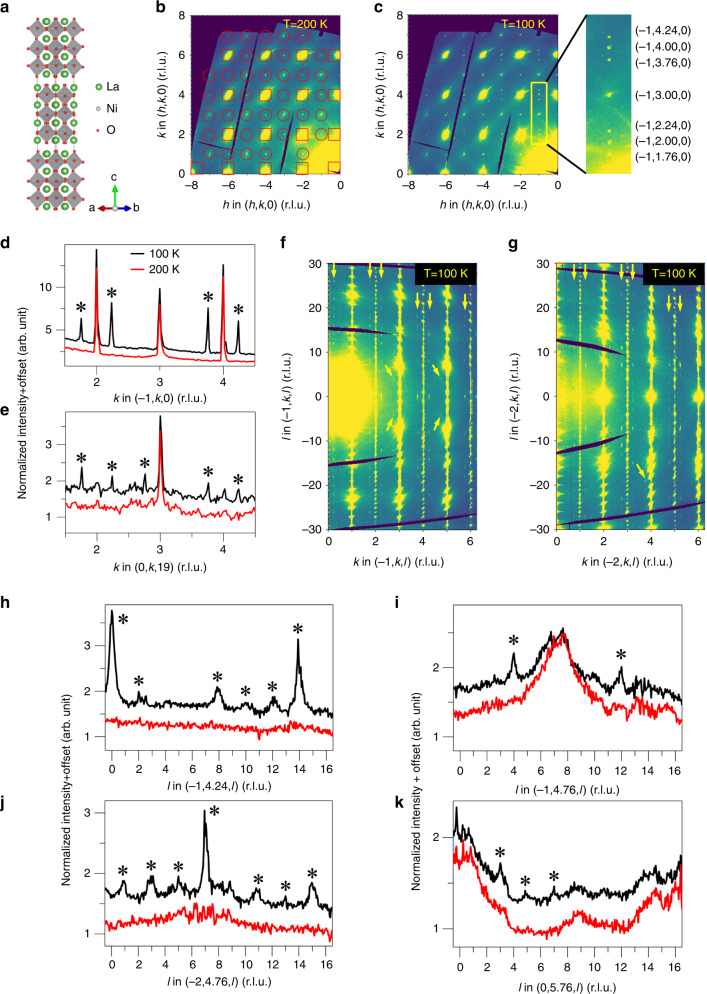
**Charge density wave order in La**_**4**_**Ni**_**3**_**O**_**10**_. **a** Crystal structure of La_4_Ni_3_O_10_. The unit cell contains two trilayer perovskite-like blocks that are related through the *B*-centering operation of the *Bmab* space group. **b** *hk*0 plane at 200 K as measured at Sector 15-ID-D. The data are integrated over 0.05 r.l.u. in *l*. Hollow squares, fundamental Bragg peaks from high symmetry *Bmab*; hollow circles, Bragg peaks resulting from monoclinic distortion to *P*2_1_/*a*. **c**, *hk*0 plane at 100 K. **d**, **e** Line cuts through data along *k* direction at 100 and 200 K. The cuts have been integrated over 0.04 r.l.u. in *h* and 0.05 r.l.u. in *l*. **f**, $\bar 1kl$ plane at 100 K. The data are integrated over 0.02 r.l.u. in *h* (*h* = −1). **g** $\bar 2kl$ plane at 100 K. **h**–**k** Line cuts through data along *l* direction at 100 and 200 K. Note red for 200 K and black for 100 K. The cuts have been integrated over 0.02 r.l.u. in *h* and 0.04 r.l.u. in *k*. In **d**, **e**, **h**–**k**, the data are shifted for clarity. Asterisks (*) mark the superlattice reflections. Yellow arrows in **f**, **g** point to superlattice reflections.

Figure 1a–c show in-plane resistivity, in-plane magnetic susceptibility, and heat capacity measured on La_4_Ni_3_O_10_ single crystals, which adopt a pseudo-orthorhombic *Bmab* structure shown in Fig. 2a (properly indexed as monoclinic, *P*2_1_/*a*, see ref. ^11^ and Supplementary Note 1). The in-plane resistivity (Fig. 1a) drops with decreasing temperature, indicating metallic behavior, and an anomaly is observed at *T*_MMT_ ≈ 140 K. We note that the temperature-dependent transport behavior of La_4_Ni_3_O_10_ near *T*_MMT_ resembles that reported for several density wave materials including chromium^19^, purple bronzes^20^, and rare-earth tritellurides^21^. The in-plane magnetic susceptibility (Fig. 1b) shows a sharp decrease at *T*_MMT_, but it does not follow either a Pauli or Curie-Weiss form above *T*_MMT_ in the temperature range measured, as it monotonically increases with increasing temperature. It does, however, resemble that of CDW materials such as the one-dimensional K_0.3_MoO_3_^20^, in which the temperature dependence of the susceptibility above the MMT was attributed to CDW fluctuations and a pseudogap in the electronic density of states^22^. The heat capacity of La_4_Ni_3_O_10_ at low temperature (inset, Fig. 1c) was fit to the typical form, *C*_*p*_/*T* = *γ* + *βT*^2^, where *γ* is the Sommerfeld coefficient and the *βT*^2^-term arises from phonons. The fit leads to *γ* = 13.3 mJ mole^−1^ K^−2^ and *β* = 0.37 mJ mole^−1^ K^−4^, which agrees with a previous measurement of *C*_*p*_ on polycrystalline samples by Wu et al.^23^ (*γ* = 13.5 mJ mole^−1^ K^−2^) and by Kumar et al.^13^ (*γ* = 15.5 mJ mole^−1^ K^−2^); however, we find a significantly larger Debye temperature (*θ*_*D*_ = 450 K) than that reported by either group (*θ*_*D*_ = 256 K^23^, 384 K^13^, respectively). For comparison, reported values for single crystal LaNiO_3_ are *γ* = 11.7 mJ mole^−1^ K^−2^ and *θ*_*D*_ = 465 K^2^. From the Sommerfeld coefficient, *γ* = π^2^*k*_*B*_^2^*N(E*_*F*_*)*/3, the density of states at the Fermi level, *N*(*E*_*F*_), in La_4_Ni_3_O_10_ is estimated to be 1.9 states eV^−1^ per Ni, significantly less than that of LaNiO_3_, 5.0 states eV^−1^ per Ni. Using the paramagnetic band structure, we find *N*(*E*_*F*_) = 2.6 states eV^−1^ per Ni, which is substantially larger than the measured value. This suppression of states at *E*_*F*_ further corroborates our inference of a pseudogap drawn from the magnetic susceptibility and transport data.

### Charge density wave order

Using single crystal synchrotron x-ray diffraction, we observed charge superlattice (SL) peaks in La_4_Ni_3_O_10_ below the MMT, suggesting the appearance of a charge-ordered state. The temperature dependence of the integrated intensity of one such SL reflection is shown in Fig. 1d. Its onset at *T*_MMT_ correlates with resistivity, magnetic susceptibility and heat capacity measurements. We note that these charge SL reflections have intensities typically 10^4^ times weaker than the strongest fundamental Bragg reflections, explaining why they have been unobserved in prior studies of polycrystalline samples.

Figure 2b, c show the *hk*0 plane in reciprocal space at 200 K and 100 K, respectively. At 200 K, strong fundamentals appearing at integers *hk*0 obey the *Bmab* reflection condition that they are both even, but additional scattering attributable to the *P*2_1_*/a* cell is weakly observed as peaks that violate this rule. As shown in Fig. 2c, sharp first order superlattice reflections appear between certain fundamentals at 100 K, located at *h* = 2*n* + 1 and *k* = 2 *m* + 1 ± *q*_c_, (*m*, *n* integers). The appearance of SLs along *k* coincides with the observation of a pronounced anomaly in the lattice parameter *b* at the MMT^11,13^. In this particular crystal, the SL reflections appear only along *k*, indicating a single domain modulation despite the near metrical equivalence of *a* and *b*, which differ by <1%. We note that several other specimens that we investigated did show the presence of orthogonal modulations expected of a multidomain state.

Line cuts along (−1, *k*, 0) and (0, *k*, 19) are shown in Fig. 2d, e, respectively. By fitting the peaks, a modulation wavevector **q**_c_ = 0.76b* was obtained (see also Supplementary Figs. 1 and 2). The width of the SL peaks is somewhat broader than that of the corresponding fundamental peaks (Supplementary Fig. 3), indicating an in-plane correlation length, *ξ*_ab _~ 100 Å. This correlation length is comparable to that reported for stripe-order in La_1.67_Sr_0.33_NiO_4_^24^ and charge order in YBa_2_Cu_3_O_6.67_^25^.

Higher harmonics of the SL are not observed (for a discussion of sensitivity of our measurement to potential harmonics, see Supplementary Note 7 and Supplementary Fig. 12), demonstrating a sinusoidal character to the CDW. Furthermore, with a nominal doping level of 1/3 in La_4_Ni_3_O_10_, the propagation vector would be expected at commensurate **q**_c_. These facts tend to rule out alternatives such as real-space charge stripes like those found in the 1/3-hole doped single layer R–P phase La_1.67_Sr_0.33_NiO_4_^24^ or square planar trilayer La_4_Ni_3_O_8_ (ref. ^9^), subject to the caveat that a locally commensurate stripe phase with *q*_*c*_ = 2/3 and a phase slip every ≈11 diagonal rows cannot be ruled out^26^. We also point out that oxygen nonstoichiometry is unlikely to be the cause of the incommensurability. The oxygen content of La- and Pr_4_Ni_3_O_10_ are within 2 and 5 parts per thousand of 10.00, respectively^11^, with La showing an oxygen deficiency and Pr an oxygen excess. Following the arguments of Yoshizawa et al. applied to La_2−*x*_Sr_*x*_NiO_2+*δ*_ (ref. ^8^), an O deficiency of ~0.08 vacancies per formula unit, far in excess of what we measure in the case of La, would be needed to shift *q*_*c*_ from 2/3 to its observed value. Furthermore, as shown in Supplementary Fig. 2b, *q*_*c*_ is consistent across three unique samples, an unlikely occurrence were oxygen stoichiometry controlling the incommensurability of the modulation.

Figure 2f, g show $\bar 1kl$ and $\bar 2kl$ planes at 100 K, and cuts along *l* are shown in Fig. 2h–k. SL peaks along *l *occur at integers obeying the selection rule that *h* + *l* is odd. This selection rule is shown in Fig. 2h, j. For *h* = 2*n* + 1, SL peaks are located at *l* = 2 *m*, while for *h* = 2*n*, SL peaks are observed at *l* = 2 *m* + 1 (*m*, *n* integers). In contrast, when *h* and *l* have the same parity, no SL reflections are observed above background. SL peaks in these figures are observed for *h, k* of the same parity (*k* is defined by the integer from which **q**_c_ = 0.76b* is measured). As shown in Fig. 2i, k, weaker SL peaks are also found for *h*, *k* of mixed parity. Another notable feature is that the intensity in these cuts along c* is largest at *l* = 0 and 14 for SL peaks associated with odd *h* and odd *k* fundamentals (Fig. 2h), but at *l* = 7 for SL peaks at even *h* and even *k* (Fig. 2j). The interval between maxima is similar to the ratio of the length of the *c* axis to the Ni–O layer separation within a trilayer (*τ* = *c/d*_Ni-Ni_ = 7.2). The intensity distribution along c* thus reflects a sinusoidal modulation of the trilayer unit structure factor with period *τ*. A model of the CDW discussed below captures these features of the diffraction pattern. Finally, the width of SL peaks along *l* is significantly broader than that of the nearby Bragg peaks, indicating a finite correlation length, *ξ*_*c*_, of the charge order. Analysis of the peak width yields *ξ*_*c*_ ≈ 0.7*c* ≈ 21 Å (Supplementary Fig. 4), verifying weak correlation of the charge order between neighboring unit cells along *c* required to generate the selection rules discussed above.

### Spin density wave order

**Fig. 3:**
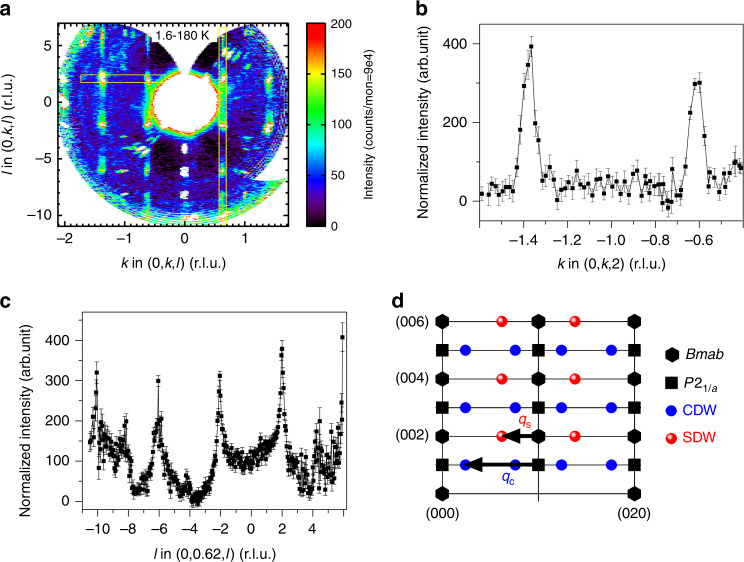
**Spin density wave order in La**_**4**_**Ni**_**3**_**O**_**10**_. **a**, 0*kl* plane with background (180 K) subtracted from 1.6 K data. **b**, **c** Line cuts along (0, *k*, 2) and (0, 0.62, *l*), respectively. **d** Schematic of the 0*kl* plane showing location of *Bmab* and *P*2_1_*/a* fundamentals and both CDW and SDW superlattice reflections. Error bars represent one standard deviation.

Single crystal neutron diffraction data were measured to test for the presence of an SDW concomitant with the CDW. If such an SDW were present, a coupling between these two density waves is expected to occur such that *q*_*c*_ = 2*q*_*s*_^27^. Figure 3a shows the 0*kl* plane in the reciprocal space of the neutron scattering data, with a background measured at 180 K subtracted (additional measurements in the *hk*0 plane are discussed in the Supplementary Note 4 and Supplementary Fig. 5). SL reflections attributed to magnetic scattering are observed at *k* = 1 ± *q*_*s*_, −1 ± *q*_*s*_. A cut along (0, *k*, 2) is presented in Fig. 3b, where magnetic SL reflections appear at *k* = −1.38 and −0.62, i.e., −1 ± *q*_*s*_. Noting that (0, 1) corresponds to the “(π, π)” antiferromagnetic wavevector that is frequently employed in the cuprate literature (i.e., with reference to an undistorted 3.8 Å × 3.8 Å tetragonal sub-cell), the SDW propagation vector in La_4_Ni_3_O_10_ was determined to be **q**_s_ = 0.62b*, that is **q**_s_ = (0, 1–*q*_*s*_, 0), with *q*_*s*_ = 0.38. With this assignment of the magnetic SL, *q*_*c*_ = 2*q*_*s*_ (Fig. 3d), as expected for a coupling between the CDW and SDW. The cut along (0, 1 − *q*_*s*_, *l*) shown in Fig. 3c evidences strong SL peaks along c* at *l* = ±2, ±6, −10, i.e., *l* = 4*n* + 2. This intensity distribution suggests antiferromagnetically coupled planes separated by *nc*/8, with *n* odd. This would occur if the inner planes were non-magnetic and the outer planes were π out of phase. However, a closer inspection shows weaker intensity appearing at *l* = ±4 and −8 as well, reflecting that the outer plane spacing is in reality *c*/3.6 instead of *c*/4.

We show in Fig. 1e the temperature dependence of the integrated intensity measured at (−1, 0.38, 0). As expected, the onset of intensity is observed at *T*_MMT_, coincident with the CDW. There is no compelling evidence in Fig. 1d, e for assigning charge or spin as the primary order parameter, and the onset temperature is the same for both within experimental resolution. In these ways, La_4_Ni_3_O_10_ behaves similarly to the related La_4_Ni_3_O_8_ system containing square planar Ni^10^.

### Density wave models and simulations

We begin with the SDW. Prominent peaks at *l* = 2 and 6 along the (0, 0.62, *l*) cut (Fig. 3c) indicate an approximate pattern of *l* = 4*n* + 2 when *h* = 0. This selection rule implies that the magnetic stacking pattern of the six planes in the unit cell (three per trilayer) is $\uparrow , - , \downarrow$;$\uparrow , - , \downarrow$, where – represents a node. This argument holds regardless of the direction of spin polarization. Had the planes been spaced precisely *d* = *c*/8 apart (as in the related compound La_4_Ni_3_O_8_^10^), then the *l* = 4*n* + 2 selection rule would be exact. We note that such a magnetic ground state, with an SDW node on the inner planes, is unusual. A related example may be layered cuprates, where the inner and outer planes differ electronically due to different effective doping levels^28^. In La_4_Ni_3_O_8_, the magnetic stacking pattern is ↑,↓,↑ instead (leading to a prominent peak at *l* = 4, which is notably absent here), with $\uparrow , - , \downarrow$ being an excited state.

**Fig. 4:**
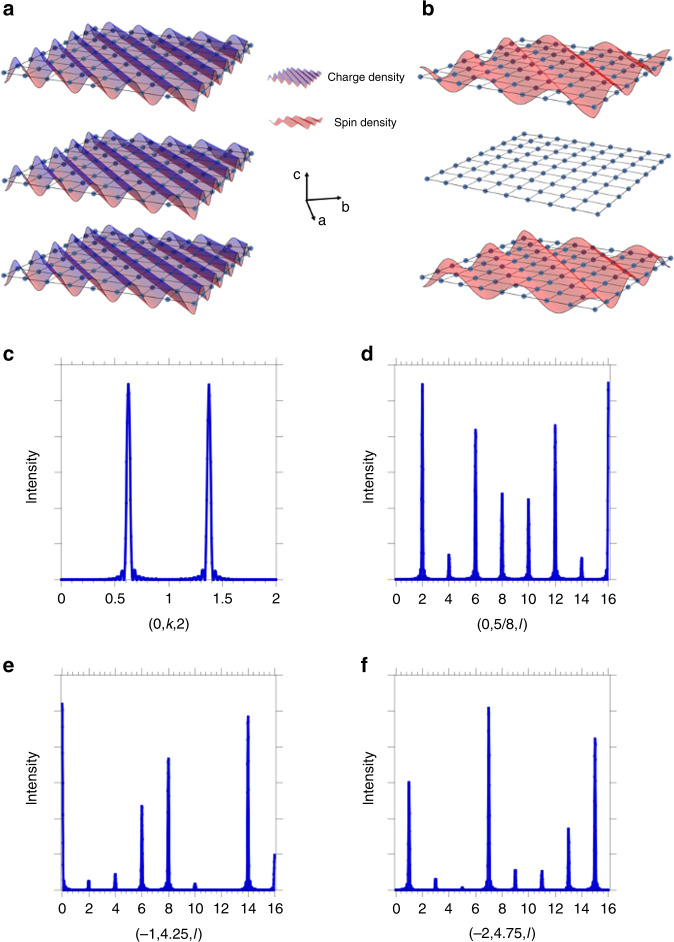
**Density wave simulations**. **a**, **b** Model for the CDW and SDW plotted in the orthorhombic *a-b* plane. One trilayer is shown. For **a**, the CDW exists on all layers and is in-phase between the layers. For **b**, the SDW has a node on the inner plane and is out-of-phase between the two outer planes. In addition, the CDW in **a** is out-of-phase between trilayers, but the SDW in **b** is in-phase. **c**, **d** Predicted SDW diffraction intensities along *k* and along *l*. **e**, **f** Predicted CDW diffraction intensities along *l*. **e** and **f** differ in having either even or odd *l* peaks.

The simplest model is to assume the in-plane behavior of the SDW has the form cos(**q**_⟂_. **r**_⟂_) with **q**_⟂ _= (0,1–*q*_*s*_) and **r**_⟂_ the in-plane coordinates of the Ni ions. Our calculations implicitly assume that the spin direction is along **a** (i.e., in the basal plane and perpendicular to **q**_s_), which is consistent with our experimental observations^11^, although additional (polarized) measurements would be required for absolute determination of the spin direction. For a given plane, the SDW corresponds to diagonal rows of aligned spins as found in La_4_Ni_3_O_8_^10^ and La_1.67_Sr_0.33_NiO_4_^27^. We choose a commensurate value of *q*_*s*_ = 3/8 to facilitate comparison to real space models. The resulting SDW pattern is shown schematically in Fig. 4b. The intensity distribution is calculated from

1 $$I\left( {\boldsymbol{k}} \right) = \left| \sum_{\mathbf{r}} {{\mathrm{c}}_{\mathrm{z}}{\mathrm{e}}^{{\mathrm{i}}{\mathbf{k}} \cdot {\mathbf{r}}}{\mathrm{cos}}\left( {{\mathbf{q}}_ \bot \cdot {\mathbf{r}}_ \bot } \right)} \right|^2$$

with *c*_*z* _= 1, 0, −1; 1, 0, −1 encoding the magnetic stacking pattern along *c* discussed above with the sum **r** over Ni atoms (normalized by their number). This expression accounts for the centering translation in the phasing between the trilayers. The resulting (0, *k*, 2) and (0, 5/8, *l*) cuts are shown in Fig. 4c, d, respectively, and should be compared to the experimental data in Fig. 3b, c, respectively. In Fig. 4d, note the small peak at *l* = 4 and the larger peaks for even values of *l* beyond *l* = 6. This arises from the fact that the planes are spaced within the trilayer by ~*c*/7.2 rather than by *c*/8. In Supplementary Fig. 7, results for different assumed SDW stackings along *c* are also shown, along with a comparison of cuts along *l* for *h* = 0 and *h* = −1 in Supplementary Fig. 8.

We now turn to the CDW peaks. Strong CDW peaks are seen for *h* + *l* = 2*n* + 1 and *k* + *l*= 2*n* + 1. This pattern, as well as the presence of a strong peak at *l* = 7 for certain momentum cuts like (0, −3.24, *l*), implies that the CDW is (1) present on all planes, (2) is in-phase within a trilayer, and (3) is out-of-phase between successive trilayers. For (1), because *d*~*c*/7.2 (explaining the strong peak at *l* = 7) and because the pattern of CDW *l* harmonics does not reflect that of the SDW discussed above, the CDW must be present on all planes. Condition (2) derives from the *Bmab* (1/2, 0, 1/2) face-centered translation reflection condition, i.e., *h* + *l* = 2*n*. This general *Bmab* condition becomes *h* + *l* = 2*n* + 1 if the modulation in successive trilayers is π out of phase. The other condition, *k* + *l* = 2*n* + 1, occurs because the Ni ion positions deviate only slightly from (00*z*). This slight deviation generates weak reflections that satisfy *k* + *l* = 2n as well. The resulting CDW pattern, modeled by ***q***_⟂ _= (0,2–*q*_*c*_) with *q*_*c*_ = 3/4 (and *c*_*z* _= 1, 1, 1; −1, −1, −1), is shown in Fig. 4a. Representative cuts demonstrating strong CDW peaks are shown in Fig. 4e, f. Note that **q**_c_ = 2**q**_s_, as expected when relating the CDW peaks to those of the SDW. Results for different assumed CDW stackings along *c*, as well as contrasting different *k* and *l* cuts, are shown in Supplementary Figs. 9–11.

The fact that a simple cosine wave can explain the data, along with the observed incommensurability and the observation that this is a metal to metal transition, all support the picture of an itinerant density wave with a 2*k*_*F*_ instability, as seen in chromium^19^. Unlike chromium, however, the CDW peaks in La_4_Ni_3_O_10_ are quite strong, and there is no evidence (Fig. 1d, e) that the CDW amplitude is secondary in the sense of the Landau theory of phase transitions (i.e., quadratic in the SDW amplitude). In this regard, La_4_Ni_3_O_10_ echoes its reduced analog, La_4_Ni_3_O_8_, though in that case one finds a semiconductor to insulator transition and a real space charge-stripe and spin-stripe scenario with a commensurate wavevector^9,10^. This behavior contrasts with chromium, where the CDW/strain wave is clearly a secondary order parameter^29^.

## Discussion

Recent reports on the electronic structure of the related trilayer R-P phases Pr_4_Ni_3_O_10_ (ref. ^12^) and Nd_4_Ni_3_O_10_ (ref. ^14^) have stressed the importance of differentiating the inner layer and outer layers of the trilayer blocks. Indeed, because of symmetry, some degree of charge differentiation between the layers is inevitable. From a DFT perspective, only minor quantitative differences (0.024 electrons) are found between the inner and outer layers. The model presented above for the CDW posits a uniform charge density modulation in all three layers, but other models we have studied with nonuniform weights only differ in a quantitative sense (Supplementary Fig. 9). We note that differential doping could offer an explanation for the node in the SDW observed on the inner layer, as suggested for multi-layered cuprates^28^, though we remark that our modeling unambiguously finds that the CDW is present on the inner plane as well.

The effect of chemical or physical pressure on the MMT in the R_4_Ni_3_O_10_ (R = La, Pr, Nd) series has recently been shown through the systematic response of *T*_MMT_ to these parameters^14,15^. The monotonic variation of the transition is consistent with a Fermi surface driven instability sensitive to variables that can modify the electronic band structure and hence the relevant nesting vector(s)^30,31^. Indeed, we find that by substituting Pr for La that the propagation vector responds to the effect of chemical pressure, albeit weakly, with *q*_*c*_ = 0.78 for Pr_4_Ni_3_O_10_, and *q*_*c*_ = 2*q*_*s*_ (Supplementary Fig. 6). In support of this nesting picture, we show calculations of the susceptibility from band theory in Supplementary Note 9 (Supplementary Fig. 13).

In some sense, one could regard La_4_Ni_3_O_10_ as a doped version of LaNiO_3_, which is a metal known to lie near an antiferromagnetic quantum critical point^32^. Indeed, La_4_Ni_3_O_10_, with an average Ni valence of 2.67 (d^7.33^), corresponds to Ni^2+^ and Ni^3+^ in a 1:2 ratio and as such is 1/3 electron-doped relative to the parent LaNiO_3_ perovskite. With its trilayer structure, the ground state of quasi-2D La_4_Ni_3_O_10_ lies at a crossover between the paramagnetic 3D metal LaNiO_3_ and the insulating, more 2D single layer nickelates, La_2-*x*_Sr_*x*_NiO_4_ (*x* < 1). Rather than the static real-space charge and spin stripes found in the latter materials when *x* < 0.5^27^, the result of this moderately strengthened coupling in La_4_Ni_3_O_10_ is an incommensurate charge-density and spin-density wave order that appears to be unprecedented in a *3d* oxide. Metallic conductivity coexisting with the CDW and SDW implies a partial gapping of the Fermi surface at *T*_MMT_. Indirect support for this latter point comes from the specific heat, where *N*(*E*_*F*_) for metallic LaNiO_3_ is nearly three times larger than that of La_4_Ni_3_O_10_. Future studies using, for instance, high resolution ARPES and tunneling could shed light on this putative gapping (The gap reported in ref. ^16^ results from orthorhombic backfolding of the 3*z*^2^-*r*^2^ bands, as can be seen near the *Γ* point in Supplementary Fig. 13a. As such, it is unrelated to the CDW/SDW gap discussed here).

We note that Jahn–Teller physics implied by the formal presence of Ni^3+^ is not important in La_4_Ni_3_O_10_, as evidenced through the suppressed orbital polarization between *x*^2^*−y*^2^ and 3*z*^2^*−r*^2^ states that we reported earlier from XAS measurements^33^. Indeed, this weak orbital polarization implies that bandwidth effects dominate over any potential Jahn-Teller orbital splitting in La_4_Ni_3_O_10_.

Finally, the relation of the incommensurate modulations observed here and the antiferromagnetic QCP identified for the perovskite LaNiO_3_ is not clear. This could be studied, for instance, by hole-doping La_4_Ni_3_O_10_ toward metallic LaNiO_3_. In a like vein, the relation of La_4_Ni_3_O_10_ to insulating La_2−*x*_Sr_*x*_NiO_4_ could be investigated by electron-doping instead, or else by studying the bilayer homolog La_3_Ni_2_O_7_. From such studies, it should be possible to build a unified understanding of this exceptional group of quantum materials.

## Methods

### Sample growth and characterization

Single crystals of La_4_Ni_3_O_10_ were grown using a vertical optical image high-pressure floating zone furnace at 20 bar pO_2_ (Model HKZ, SciDre, Germany (The identification of any commercial product or trade name does not imply endorsement or recommendation by the National Institute of Standards and Technology))^11^. Precursor powders of La_4_Ni_3_O_10_ were hydrostatically pressed into polycrystalline rods (length = 100 mm, diameter = 8 mm) and sintered for 24 h at 1400 °C. La_4_Ni_3_O_10_ crystals were grown directly from the sintered rod at pO_2_ = 20 bar using a 3-kW xenon arc lamp to heat the zone. A similar procedure was applied to Pr_4_Ni_3_O_10_, but sintering at 1100 °C was necessary to avoid cracking of the rod. Pr_4_Ni_3_O_10_ crystals were grown at pO_2_ = 140 bar through two steps using a 5-kW xenon arc lamp: a fast pass (30–50 mm/h) followed by growth at the same pressure at 5 mm/h. During each growth, a flow rate of 0.1 l/min of oxygen was maintained. Feed and seed rods were counter-rotated at 27 and 20 rpm, respectively.

### Magnetic susceptibility

Magnetic susceptibility measurements were performed on single crystals using a Quantum Design MPMS3 SQUID magnetometer (The identification of any commercial product or trade name does not imply endorsement or recommendation by the National Institute of Standards and Technology). Specimens were attached to a quartz holder using a minute amount of glue. ZFC-W (Zero-field cooling with data collected on warming), FC-C (field cooling with data collected on cooling) and FC-W (field cooling with data collected on warming) data with magnetic field **H∥**ab were collected between 1.8 and 300 K under an external field of 0.4 T. The out-of-plane magnetic susceptibility is discussed in a separate paper^11^. In the ZFC-W protocol the sample was cooled in zero field to 10 K at a rate of 35 K/min and then to 1.8 K at a rate of 2 K/min, and DC magnetization recorded on warming (2 K/min). In the FC-C and FC-W protocols, the magnetization was recorded (2 K/min) in a fixed field of 0.4 T.

### Electrical resistivity

The resistivity was measured using a four-probe method with contacts made by depositing gold pads. The temperature was controlled using the Quantum Design PPMS in the temperature range of 1.8−300 K.

### Heat capacity

Heat capacity measurements were performed on a Quantum Design PPMS (The identification of any commercial product or trade name does not imply endorsement or recommendation by the National Institute of Standards and Technology) in the temperature range of 1.8–300 K. Apiezon-N (The Identification of any commercial product or trade name does not imply endorsement or recommendation by the National Institute of Standards and Technology) vacuum grease was employed to fix crystals to the sapphire sample platform. The specific heat contribution from sample holder platform and grease was determined before mounting sample and subtracted from the total heat capacity.

### X-ray diffraction

Synchrotron X-ray single crystal diffraction measurements were performed at beamline 15-ID-D at ChemMatCARS (University of Chicago) at the Advanced Photon Source, Argonne National Laboratory. Data were collected with a 1 M Pilatus area detector using synchrotron radiation (λ = 0.41328 Å) at 100, 110, 120, 125, 127, 129, 131, 133, 135, 137, 139, 145, 200 K with temperature controlled by flowing nitrogen gas. To cover a sufficient volume of reciprocal space, ω and φ scans were used, with 1800 frames (0.2 deg/frame, continuous scan) collected at each ω angle. Several single crystals were checked in this experiment, and the SL peaks are reproducible. The 3D reciprocal space volumes were generated from the data using the CCTW package^34^ and visualized using NeXpy^35^, which was used to produce the linecuts. To obtain the precise wavevector, data were collected at beamline 33-BM-C^36^ (Advanced Photon Source) using a point detector. The wavevector **q**_c _= 0.76b* was obtained by fitting the peaks.

### Neutron elastic scattering

Unpolarized measurements in the *hk*0 and 0*kl* scattering planes were performed on the MACS cold neutron multi-axis spectrometer at the NIST Center for Neutron Research (NCNR) with λ = 4.05 Å. Four crystals were coaligned for the *hk*0, and a crystal of mass ≈40 mg was used for 0*kl* plane, and data were collected at 1.6 and 180 K. The SDW order parameter was determined by following the evolution of the intensity at (−1, 0.38, 0) on warming.

### Density wave models and simulations

For numerical purposes, the intensities were calculated using 10 unit cells along *c* (20 trilayers) and 25^2^ orthorhombic unit cells in the plane (for a total of 75000 Ni atoms). The slight *Bmab* deviations of the outer plane Ni ions from their *I4/mmm* positions were taken into account, using atomic coordinates given in ref. ^11^ Details are given in the Supplementary Note 6 (Supplementary Figs. 7–11) along with real space stripe simulations in Supplementary Note 7 (Supplementary Fig. 12).

### Computational details

We performed first-principles calculations using the WIEN2k^37^ code with the Perdew–Burk–Ernzerhof^38^ version of the generalized gradient approximation. A Fourier series spline fit^39^ to the bands was made with 2736 face centered orthorhombic Fourier functions fit to 448 *k* points in the irreducible wedge of the Brillouin zone. Both the density of states and the susceptibility were calculated using a tetrahedron decomposition of the Brillouin zone^40^ (3 × 8^n^ tetrahedra in the irreducible wedge with *n* = 6 used for the density of states and *n* = 5 for the susceptibility). These results are presented in Supplementary Note 9 (Supplementary Fig. 13).

## Supplementary information

Supplementary Information

## Data Availability

The data that support the findings of this study are available within the article or from the corresponding author upon reasonable request.

## Code availability

Access to the codes used to calculate density waves and electronic susceptibility will be made available by the corresponding author upon reasonable request.
