# Supplementary material for: Intertwined density waves in a metallic nickelate
Source: Nat Commun. 2020 Nov 26;11:6003. doi: 10.1038/s41467-020-19836-0 (PMC7691989; doi:10.1038/s41467-020-19836-0)
Supplement: Supplementary file 1 — Supplementary Information [file 41467_2020_19836_MOESM1_ESM.pdf]

Supplementary Notes for:

Intertwined density waves in a metallic nickelate

Junjie Zhang[1,2]*, D. Phelan[1], A. S. Botana,[3] Yu-Sheng Chen[4], Hong Zheng[1], M. Krogstad[1], Suyin Grass Wang[4], Yiming Qiu[5], J. A. Rodriguez-Rivera[5,6], R. Osborn[1], S. Rosenkranz[1], M. R. Norman[1] and J. F. Mitchell[1]*

[1]Materials Science Division, Argonne National Laboratory, Lemont, Illinois 60439, United States.
[2]Institute of Crystal Materials & State Key Laboratory of Crystal Materials, Shandong University, Jinan, Shandong 250100, China.
[3]Department of Physics, Arizona State University, Tempe, Arizona 85287, United States.
[4]ChemMatCARS, The University of Chicago, Lemont, Illinois 60439, United States.
[5]NIST Center for Neutron Research, National Institute of Standards and Technology, Gaithersburg, Maryland 20899, United States.
[6]Department of Materials Science, University of Maryland, College Park, Maryland 20742, United States.

*e-mail: junjie@sdu.edu.cn; mitchell@anl.gov

1. Crystal Structure of $La_4Ni_3O_{10}$
2. Determination of CDW propagation vector
3. Correlation length of CDW
4. Neutron scattering in the *hk*0 plane
5. CDW in $Pr_4Ni_3O_{10}$
6. Modeling of the SDW and CDW
7. Real space spin-stripe model calculation
8. Why the CDW is not on the oxygen sites
9. Band structure and Fermi surface nesting

1. Crystal Structure of $La_4Ni_3O_{10}$

The unit cell of $La_4Ni_3O_{10}$ contains two trilayer perovskite slabs of corner-sharing NiO_6 octahedra separated by a rocksalt layer of LaO (see Fig. 2a of main text). Depending on details of the growth conditions and post-growth treatment[1], $La_4Ni_3O_{10}$ crystallizes in either an orthorhombic (*Bmab*) or monoclinic ($P2_1/a$) crystal structure above T_{MMT}. The crystal studied here shows T_{MMT} = 140 K, indicating that the high temperature structure belongs to the monoclinic space group $P2_1/a$. However, this structure is pseudo-orthorhombic, and for ease of notation we employ the *Bmab* setting with lattice parameters of a~5.41 Å, b~5.46 Å, and c~27.97 Å throughout this report. In this setting, the two trilayers are symmetry-related by the *B*-centering operation.

2. Determination of CDW propagation vector

Linecuts from the 15-ID-D data were used to determine the propagation vector by fitting the SL peaks at (-1, 1.76, 0), (-1, 2.24, 0), (-1, 3.76, 0) and (-1, 4.24, 0) as well as their nearby main Bragg peaks (-1, 2, 0) and (-1, 4, 0) using a Gaussian function. A typical fit is shown in Supplementary Fig. 1. The obtained values from the Gaussian fit are listed below.

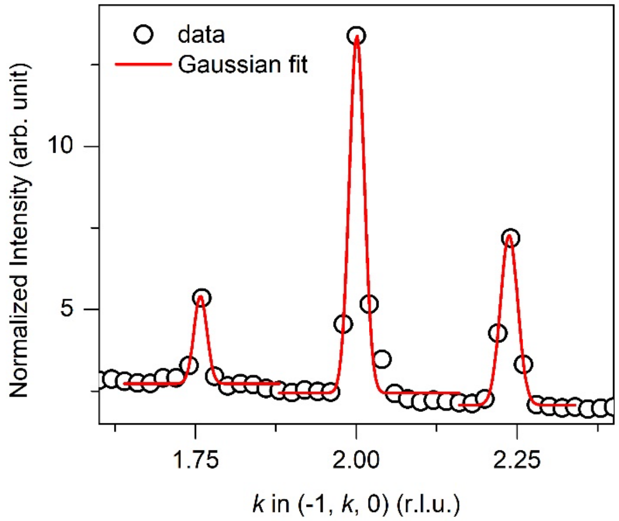

k in (-1, *k*, 0)	Value from Gaussian fit
1.76	1.76(1)
2	2.00(1)
2.24	2.24(1)
3.76	3.76(1)
4	4.00(1)
4.24	4.23(1)

Supplementary Fig. 1 Gaussian fit to the main Bragg peak (-1, 2, 0) and SLs.

Considering the limited pixels for each peak from linecuts from 15-ID-D data, we further performed single crystal diffuse scattering experiments at Beamline 33-BM-C at the Advanced Photon Source using a point detector at 70 K (λ=0.7749 Å). *hkl* scans were performed using steps of 0.0025 reciprocal lattice unit (r.l.u.) for *k* scans and 0.01 r.l.u. for *l* scans. Superlattice peaks were observed and fit to the Gaussian function (see Supplementary Fig. 2a). The obtained wavevector is (2.235-1.753)/2=0.241. The CDW wavevector is calculated from the Bragg peak (130) or (150), thus the wavevector is 0.76**b*.**

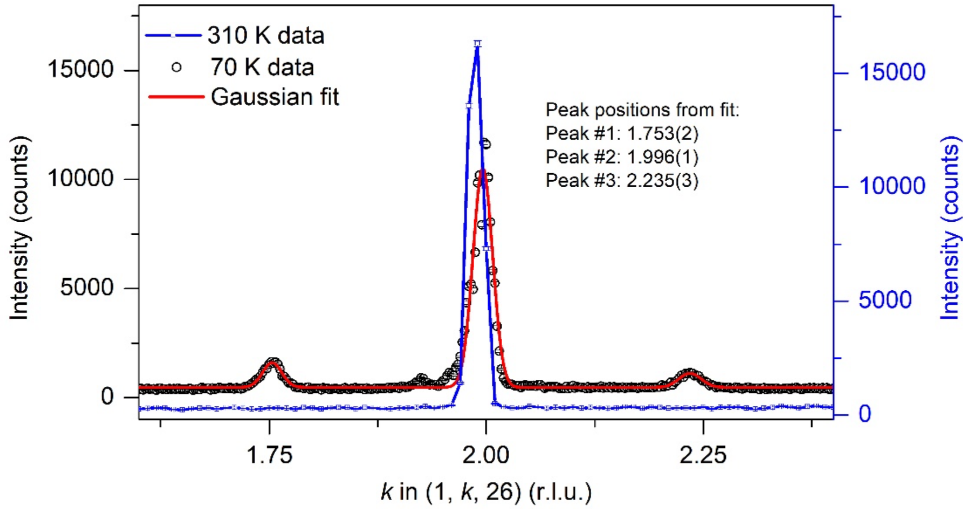

Supplementary Fig. 2a Gaussian fit to the Bragg and SL peaks of $La_4Ni_3O_{10}$.

Supplementary Fig. 2b shows x-ray scans collected on three unique single crystal $La_4Ni_3O_{10}$ specimens to establish the consistency of the charge q-vector. This consistency tends to rule out incommensurability driven by defects, disorder, or nonstoichiometry. Further evidence is presented in the main text that argues against oxygen nonstoichiometry based on the amount needed to shift the propagation vector from the commensurate q_c=2/3 expected for the nominal hole concentration to the observed incommensurate position. We estimate an O vacancy concentration ~4 times larger than measured would be required.

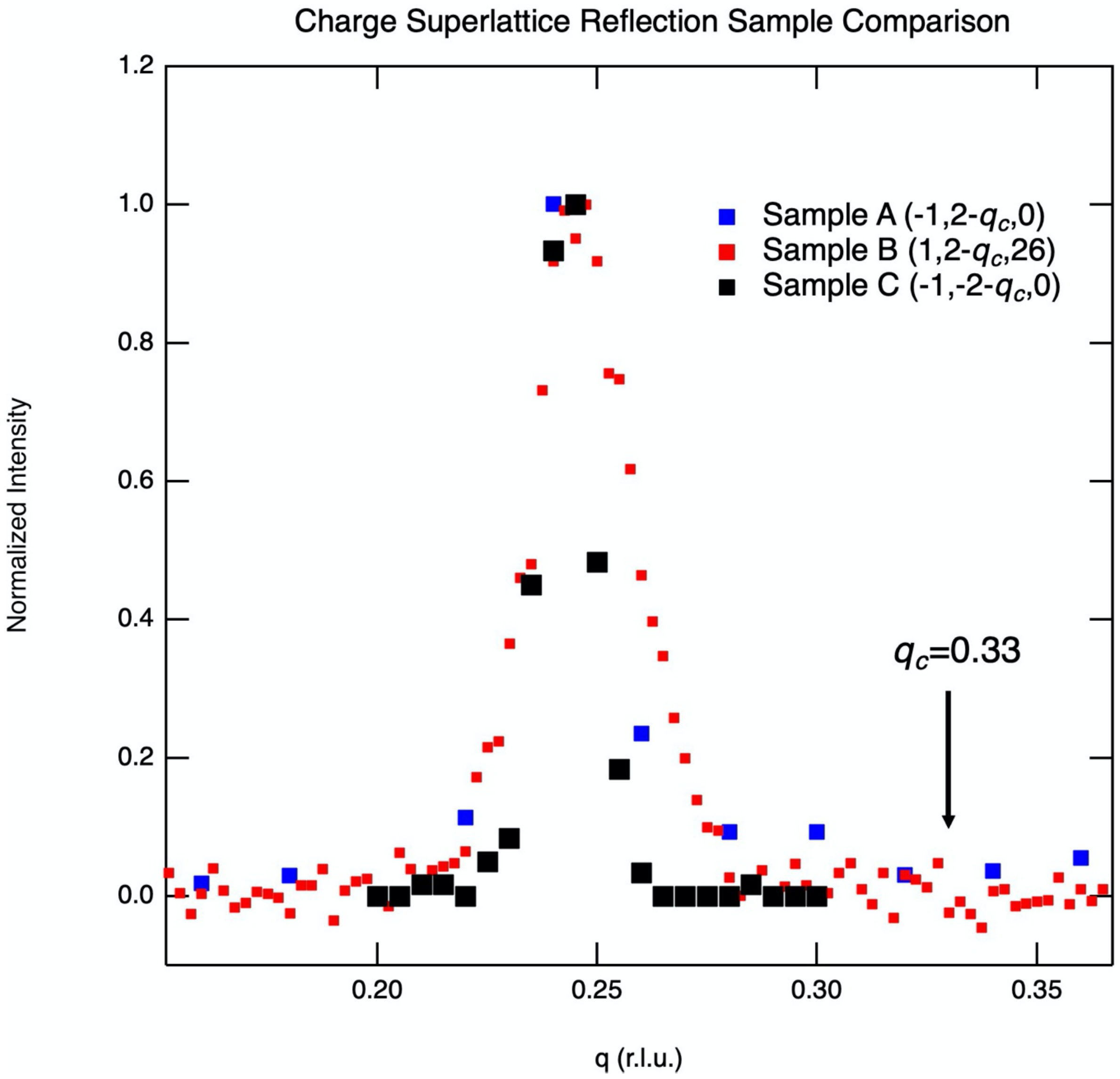

Supplementary Fig. 2b Comparison of charge q_c-vector in three unique specimens. Sample A was measured at APS Beamline 15-ID-D, Sample B was measured at APS Beamline 33-BM-C, and Sample C was measured at APS Beamline 30-ID-C. The commensurate q_c=1/3 position expected based on charge balance in a real-space stripe is marked.

3. Correlation length of CDW

Using data collected at the 33-BM-C beamline of the Advanced Photon Source, the correlation length of the charge order modulation was estimated through analysis of the peak width of the superlattice reflections. The correlation length ξ was determined as $\xi = 1/\Gamma$, where Γ is the half-width at half-maximum of the Lorentzian component. A Gaussian fit was performed on the main Bragg peak (1,4,18) in order to estimate the instrumental resolution, and this Gaussian width was fixed in the Voigt function (a convolution of Gaussian and Lorentzian functions) used to fit the superlattice peaks (Supplementary Fig. 3).

The in-plane and out-of-plane correlation length are ξ_{ab}=16-22b (87-118 Å) and ξ_c=0.67-0.87c (18.8-24 Å), respectively. The estimated correlation length along c is comparable to the 21 Å that was found independently from 15-ID-D data shown in Supplementary Fig. 4.

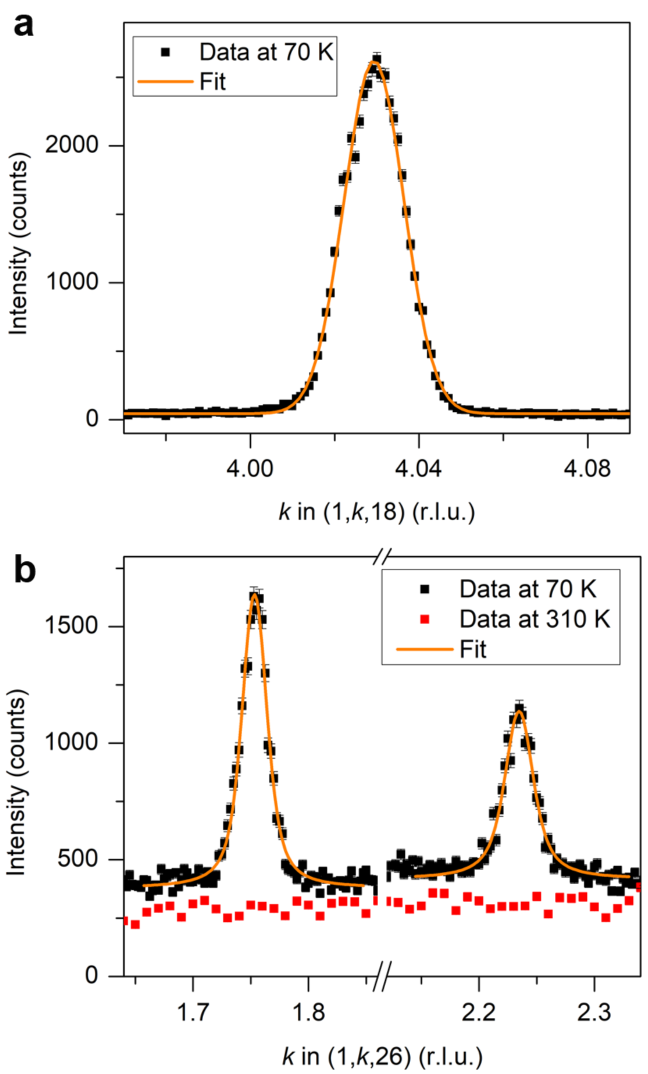

Supplementary Fig. 3 Correlation length of the CDW estimated from data collected at Beamline 33-BM-C at the Advanced Photon Source.

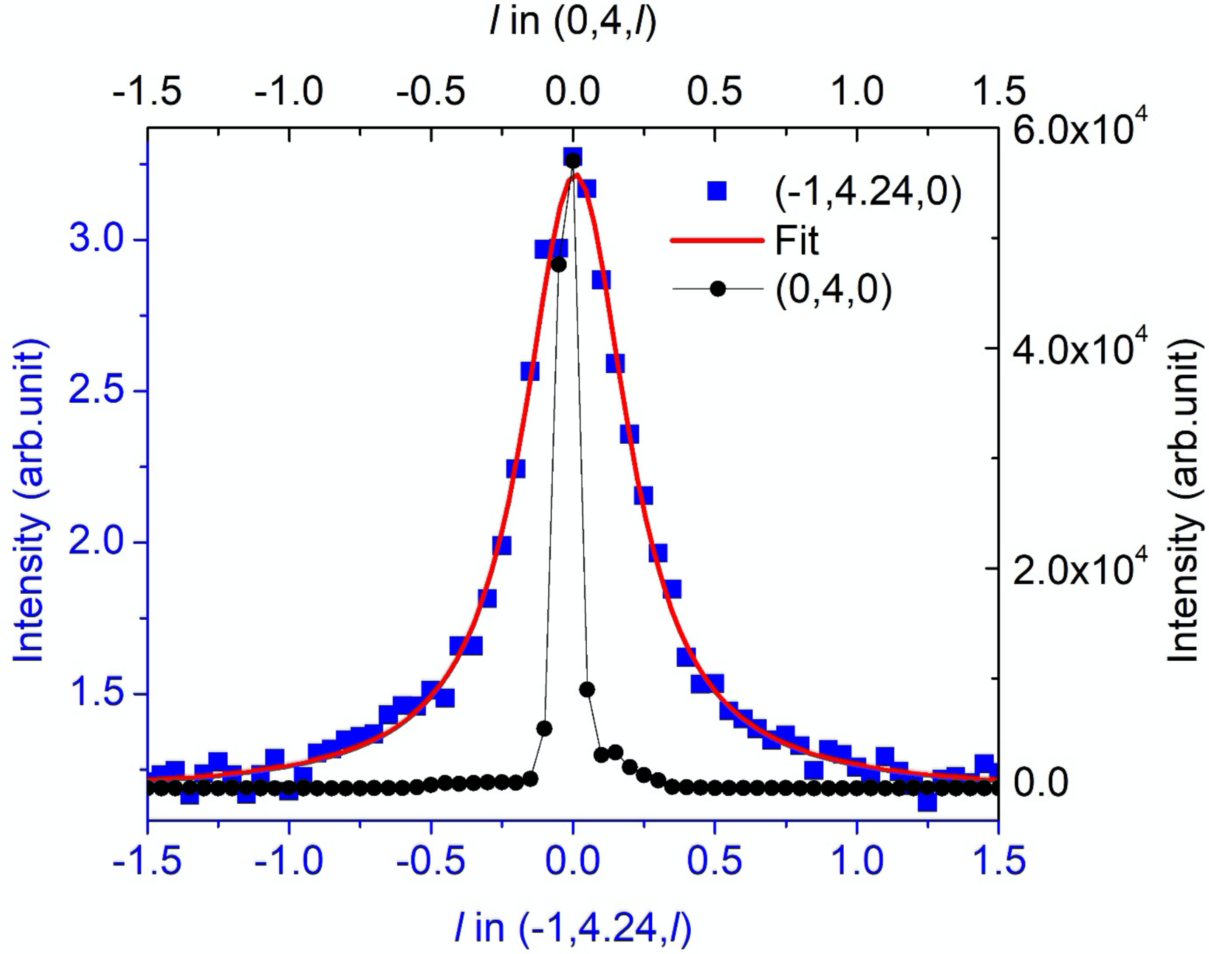

Supplementary Fig. 4 Correlation length of the CDW along the *c* direction (linecuts of 15-ID-D data). The correlation length ξ was determined as $\xi = 1/\Gamma$, where Γ is the half-width at half-maximum of the Lorentzian component. A Gaussian fit was performed on the main Bragg peak (0,4,0) in order to estimate the instrumental resolution, and this Gaussian width was fixed in the Voigt function (a convolution of Gaussian and Lorentzian functions) used to fit the superlattice peak (-1,4.24,0). Using this procedure, Γ was determined to be 0.215 r.l.u. (where 1 r.l.u.=$2\pi/c$) yielding ξ= 0.7c=21 Å.

4. Neutron scattering in the *hk*0 plane

In the main text, neutron scattering measurements were shown in the 0*kl* plane. Here, we show measurements performed in the *hk*0 plane on the same instrument. Please note that whereas the measurements in the 0*kl* plane were performed with one specimen, the measurements in the *hk*0 plane were performed with four co-aligned specimens. A 180 K data set (Supplementary Fig. 5b) has been subtracted from the 1.6 K data set (Supplementary Fig. 5a) to reveal the magnetic superlattice peaks from the noise in the raw data. Supplementary Fig. 5c shows the map with the high temperature subtracted as a background. Supplementary Fig. 5d shows a line-cut through the temperature subtracted data along *h*. Note that the signal in this plane is much weaker than the maximal signal in the 0*kl* plane because the strong maxima occur at *l*=2 and 6, rather than 0. Nevertheless, a contribution to the *hk*0 plane is observed that may result from intrinsically broad peaks in *l* that are out of plane, exacerbated by the course out-of-plane resolution of the spectrometer.

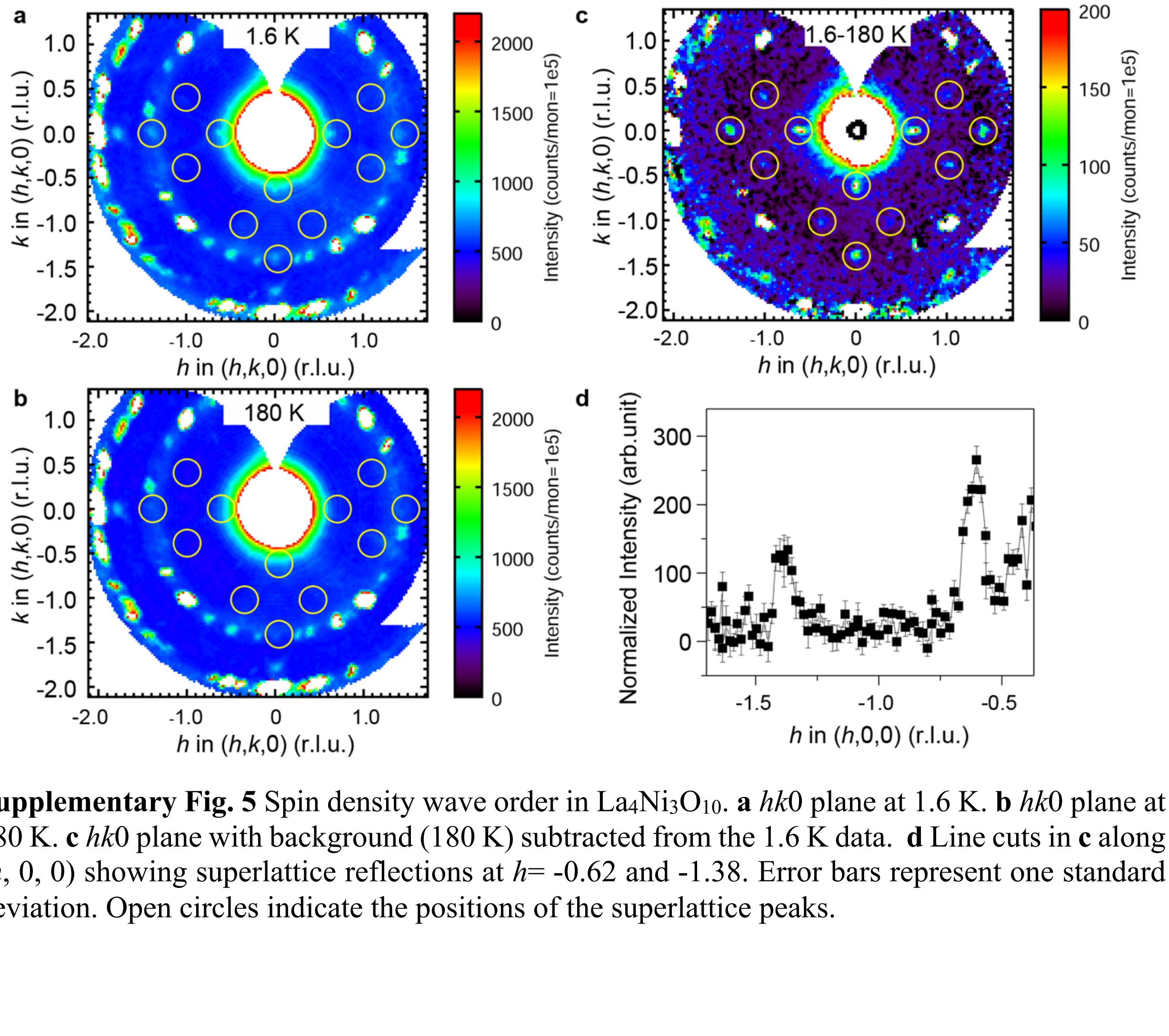

Supplementary Fig. 5 Spin density wave order in $La_4Ni_3O_{10}$. **a** *hk*0 plane at 1.6 K. **b** *hk*0 plane at 180 K. **c** *hk*0 plane with background (180 K) subtracted from the 1.6 K data. **d** Line cuts in **c** along (*h*, 0, 0) showing superlattice reflections at *h*= -0.62 and -1.38. Error bars represent one standard deviation. Open circles indicate the positions of the superlattice peaks.

5. CDW in $Pr_4Ni_3O_{10}$

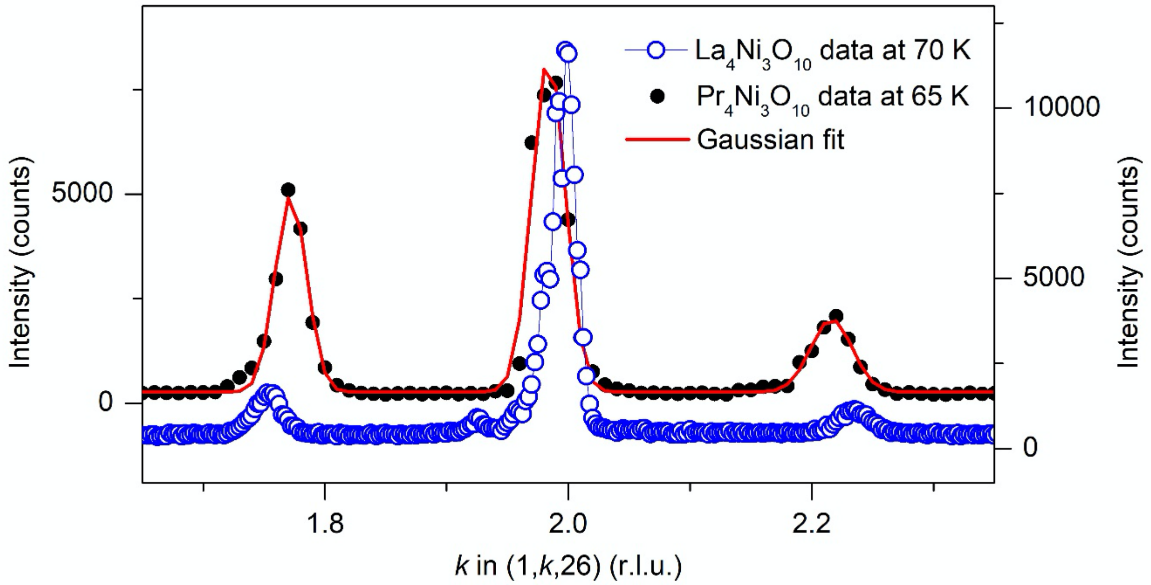

Supplementary Fig. 6 Propagation vector of the CDW in $Pr_4Ni_3O_{10}$. The peaks were fit using a Gaussian function. The obtained positions are *k*=1.772, 1.984 and 2.216, so the wavevector is 1-(2.216-1.772)/2=0.778 along **b***. Note this wavevector (0.78) is slightly different from that of $La_4Ni_3O_{10}$ (0.76).

6. Modeling of the SDW and CDW

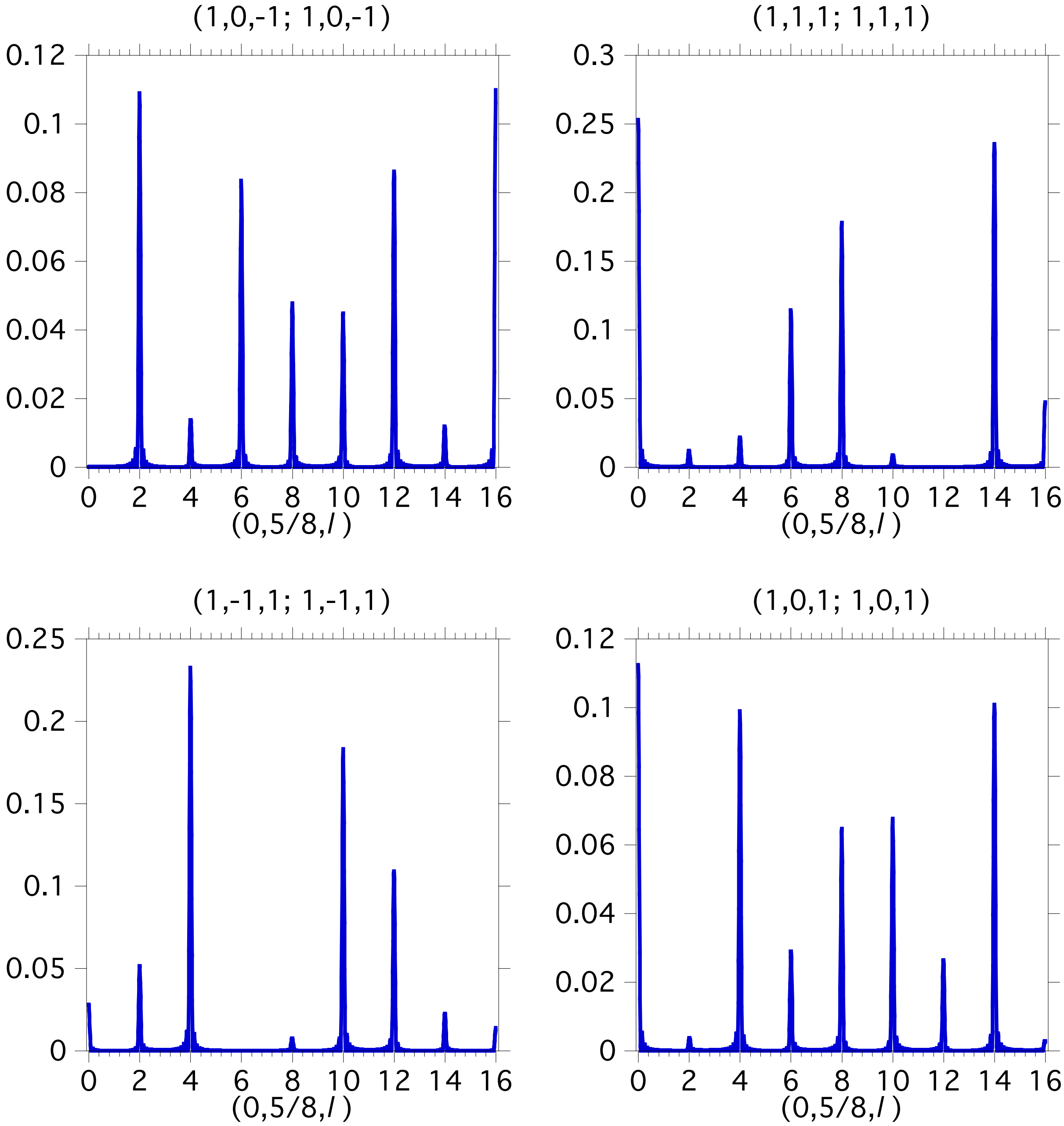

Supplementary Fig. 7 Intensities for different assumed SDW stackings along the *c*-axis for the (0, 5/8, *l*) line cut, calculated using the equation listed in the main text. The titles indicate the stacking pattern along *c* (the c_z weights of the main text). The upper left plot is the SDW model presented in the main text, which has prominent peaks at *l*=2 and 6.

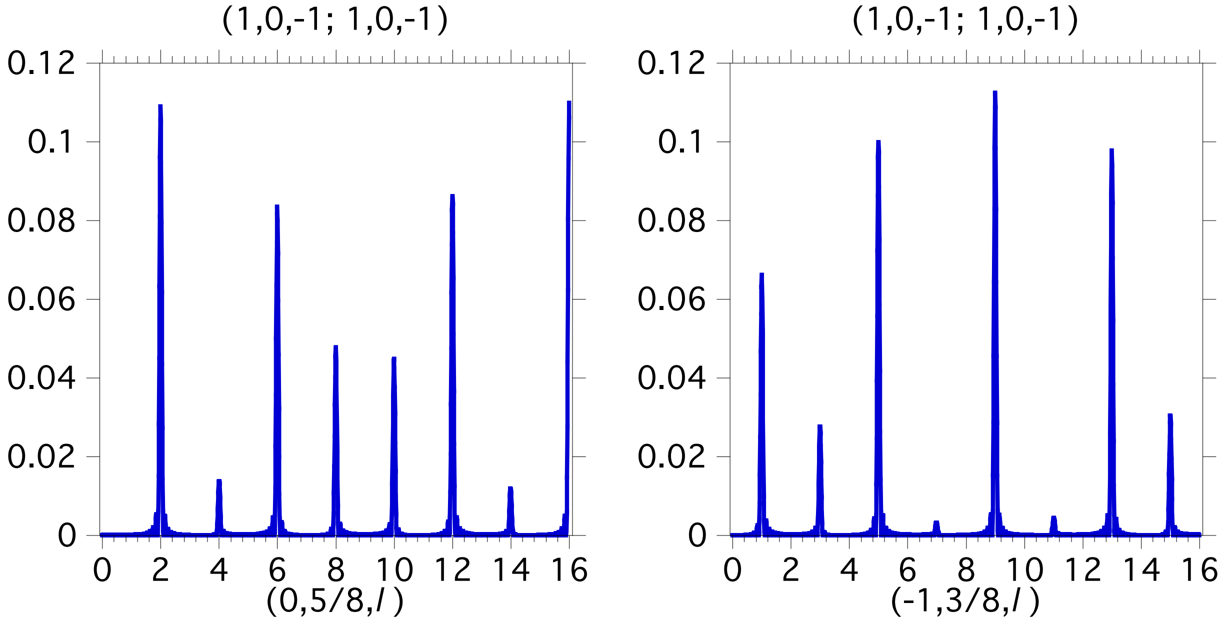

Supplementary Fig. 8 Contrasting the (0, 5/8, *l*) line cut with the (-1, 3/8, *l*) line cut for the SDW model presented in the main text.

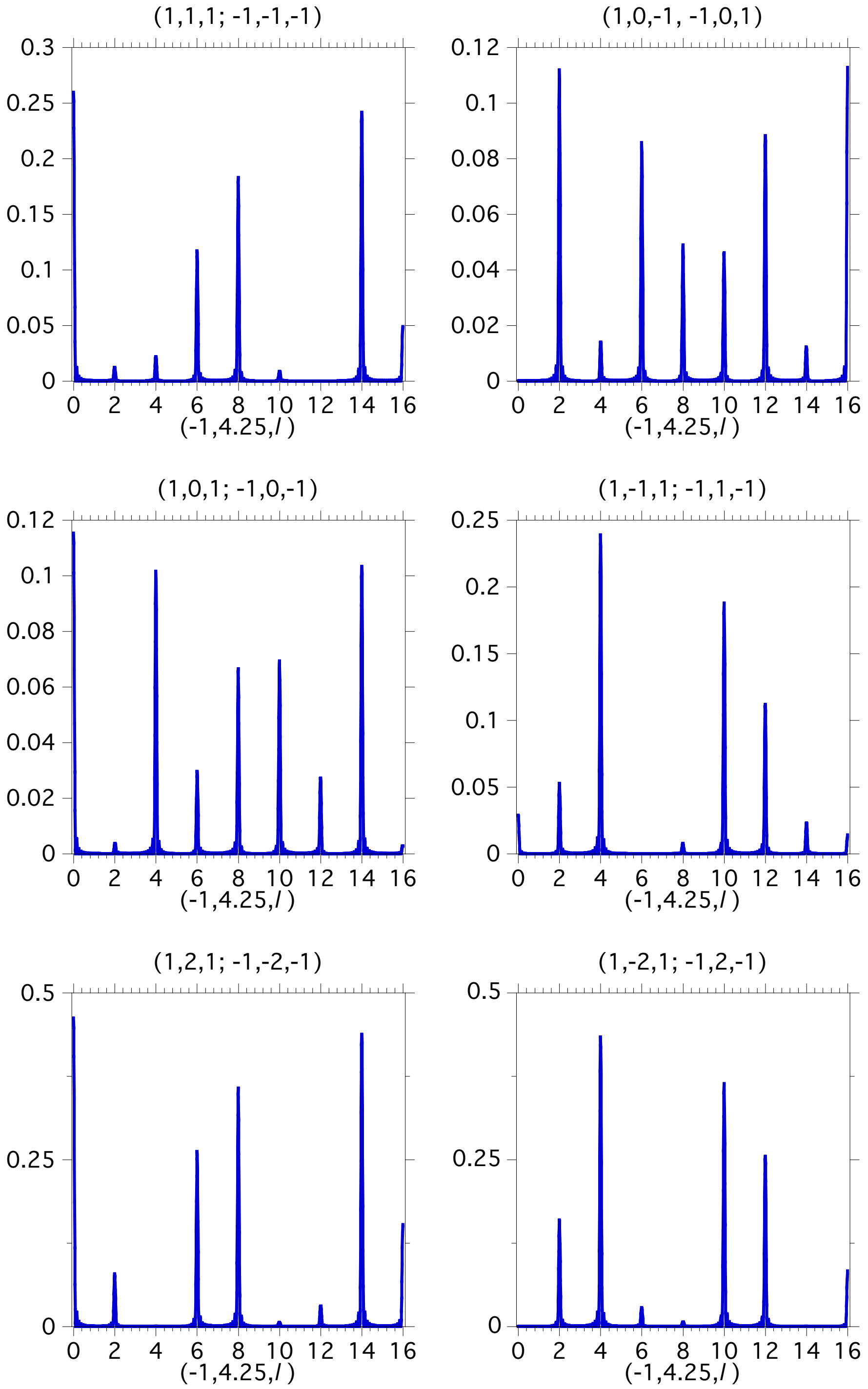

Supplementary Fig. 9 Intensities for different assumed CDW stackings along the *c*-axis for the (-1, 4.25, *l*) line cut. The titles represent the stacking patterns along ***c*** (numbers in parentheses are the c_z weights described in the main text). The upper left plot is the CDW model presented in the main text, which has prominent peaks at *l*=0 and 14.

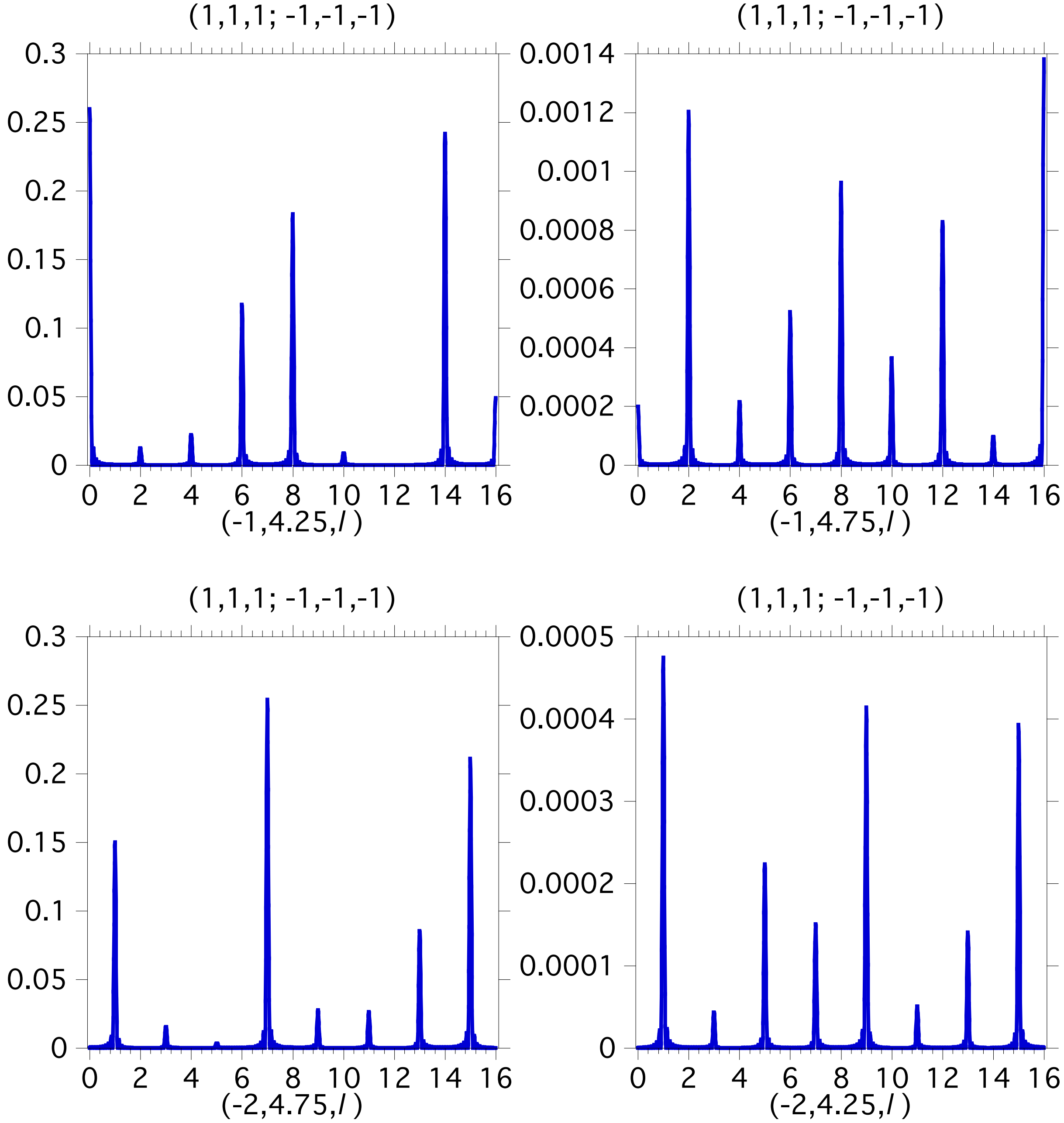

Supplementary Fig. 10 Intensities for various line cuts for the CDW model presented in the main text, contrasting strong CDW peaks (left plots) from weak CDW peaks (right plots). The latter intensities are sensitive to the *Bmab* deviations of the Ni atoms from their *I*4/*mmm* positions, and so these intensities should be further enhanced in the monoclinic phase (not modeled here).

Above we have presented a simple model that accounts qualitatively for the distribution of intensity in the charge peaks measured by XRD. There are certain systematic discrepancies in the intensity distributions that highlight the need for a more sophisticated model. For example, as shown in Supplementary Fig. 11, our model for the (-1,*k*,0) cut of main text Fig. 2d finds strong peaks at $k \pm q_c$ (*k* odd) in agreement with the data. For the (0,*k*,19) cut of main text Fig. 2e, there are significant differences. In particular, our model indicates strong peaks (*k* even) and weak peaks (*k* odd), whereas the data find these peaks to be of comparable intensity. Moreover, the SL intensities are reduced relative to those in main text Fig. 2d by 10^2 as compared to about 10^1 found in experiment. To successfully reproduce the data, one would need a more sophisticated model than the present one. For instance, taking into account the monoclinic distortion is non-trivial in the model used here since $\beta \neq 90$ degrees, meaning the simple cosine function would be de-phased when going from one layer to the next. Moreover, for hard x-rays, one is most sensitive to the atomic displacements. A strain wave model based on displacements of La, Ni, and O ions is a natural next step, albeit an involved endeavor, as can be seen from studies in the cuprates[2].

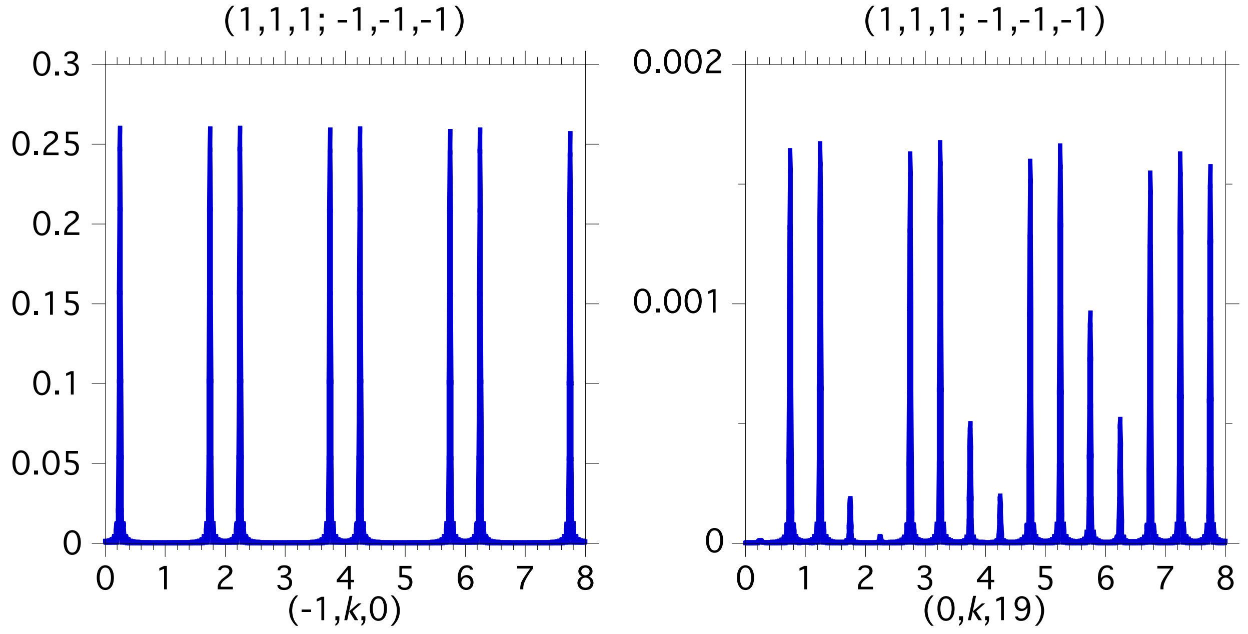

Supplementary Fig. 11 Intensities from the CDW model presented in the main text for the two *k* cuts shown in Fig. 2. For the right plot, note the alternation of strong peaks (*k* even) with weak peaks (*k* odd), in contrast to the data where these peaks have comparable intensity.

7. Real space spin- and charge-stripe model calculations

Various real space models for the density waves can be constructed by assuming 16 Ni ions per plane per magnetic unit cell. With an appropriate pattern of spins (d^7) and holes (d^8), the primary peak at 1-q_s=5/8 can be generated, but because of the real space (square wave) form, (odd) harmonics appear with the strongest at 2-3q_s=7/8, albeit with an intensity reduced by an order of magnitude relative to 1-q_s =5/8 (Supplementary Fig. 12, top). These harmonics are even larger (~17% of the fundamental) for the charge-stripe (Supplementary Fig. 12, bottom left) and would not escape our observation in the data along (-1,k,0) shown in Fig. 2d of the main text. Given the absence of these harmonics in the data and the fact that the real space pattern does not correspond to the correct doping relative to $LaNiO_3$ (which would imply a 1-q_s=2/3 instead), we did not pursue real space models further.

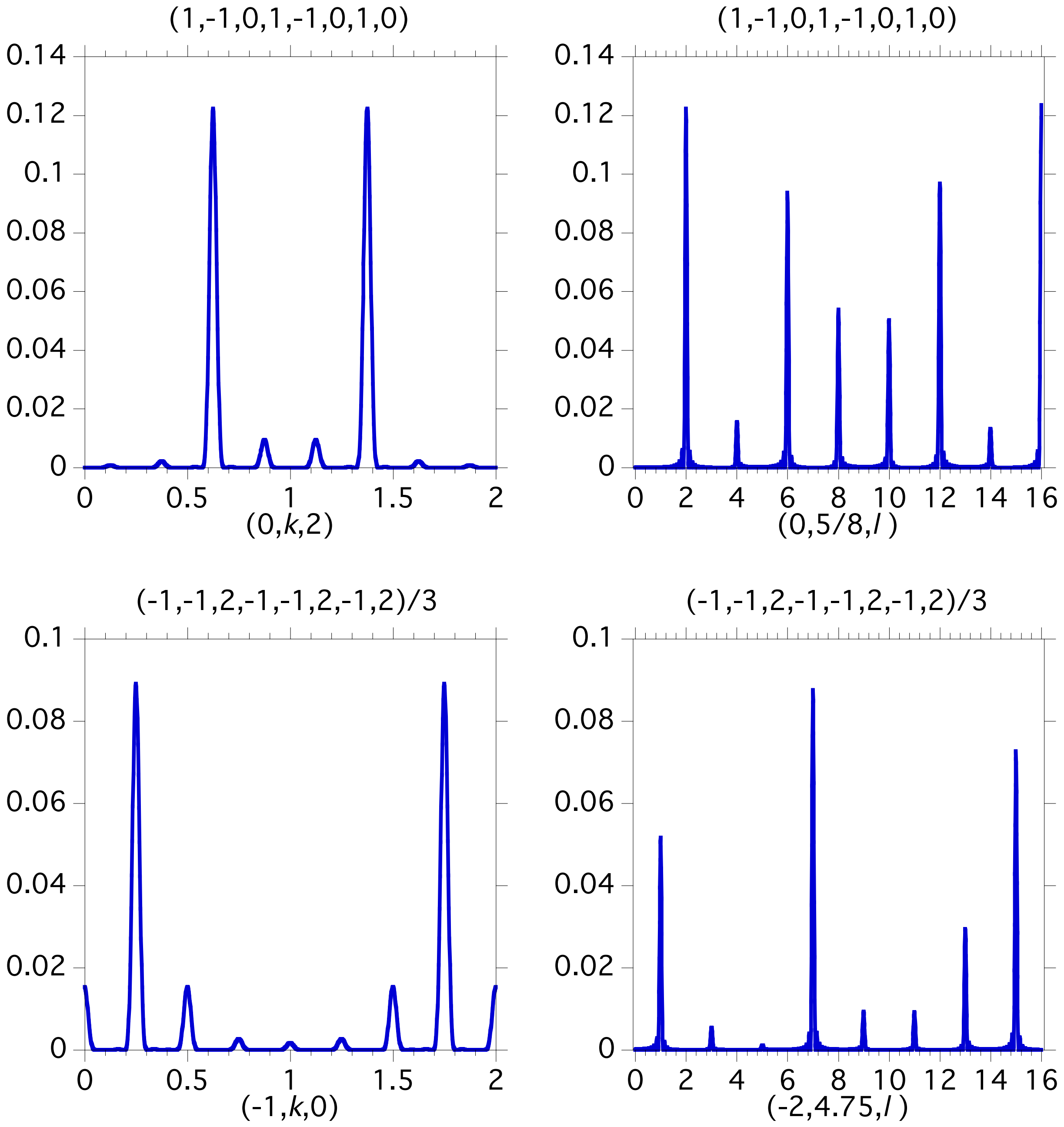

Supplementary Fig. 12 Intensities from a real space stripe model. For the top row, the title of the plots shows the SDW weights on each Ni ion for eight successive diagonal rows (±1 corresponding to d^7, 0 to d^8). For the eight rows beyond this, the spins are reversed, with the pattern repeated. The stacking along *c* is the same as before (↑, −, ↓), with the lower and upper trilayers of a given unit cell being in-phase. This acts to maintain peaks at even *l*. Since there are five non-zero weights for each eight rows, this gives rise to a primary peak at 1-q_s=5/8. The secondary peaks (*k*=7/8, etc.) are odd harmonics due to the “square wave” nature of the stripe, as compared to the sinusoidal

behavior of the itinerant model. For the bottom row, the title of the plots shows the CDW weights on each Ni ion for 8 diagonal rows (d^7 being –1/3, d^8 being +2/3, relative to the nominal valence). As with the itinerant model, the CDW is present on all planes, is in-phase between the three layers, and is out-of-phase between the trilayers. In both spin and charge cases, the BCT shift is taken into account by shifting the pattern by one place when going from the lower to the upper trilayer. Note for the charge stripe, the harmonics in the plane (k=3/2, etc.) are even larger than the spin harmonics (intensity is roughly 17% of the fundamentals at k=7/4, etc.).

8. Why the CDW is not on the oxygen sites

Our model assumes the density wave is centered on the Ni sites. This is sensible for the SDW, but what about for the CDW? A simple argument shows that the CDW cannot involve the planar oxygen sites. The in-plane planar oxygen coordinates (*Bmab*) are (¼,¼), (¾,¼), (¼,¾), and (¾,¾). For the first pair at y=1/4, the CDW will have the same amplitude, and similarly for the second pair at y=3/4. Therefore, for h odd, the sum over a pair vanishes because of the $e^{i\boldsymbol{k}\cdot\boldsymbol{r}}$ structure factor, since their x coordinates differ by ½. This is in clear contradiction to experiment. If instead one assumes a d-wave form factor for the CDW, where the CDW amplitude has opposite phase on the two members of the pair, then the sum vanishes for h even, again inconsistent with the data. We argue that the small monoclinic distortion found in $La_4Ni_3O_{10}$[1] can only cause weak violations of these rules. Finally, we remark that high energy x-rays are primarily sensitive to atomic displacements. Not only would such a strain wave differ from the CDW itself, but also other ions, such as the apical oxygens and La, can contribute, as seen in cuprates[2]. More information than we have at present would be needed to model such a complicated strain wave.

9. Band structure and Fermi surface nesting

Given the above context, we can ask whether Fermi surface nesting is relevant to the physics of $La_4Ni_3O_{10}$, as demonstrated in chromium[3] and suggested as well for $LaNiO_3$[4]. A recent ARPES study on $La_4Ni_3O_{10}$[5] suggests that the opening of a gap in the $3z^2$-r^2 band accompanies the CDW at T_{MMT}. We note that $La_4Ni_3O_{10}$ crystallizes in an orthorhombic (*Bmab*) or monoclinic ($P2_1/a$) supercell of the tetragonal *I*4/*mmm* parent, leading to backfolding of the bands. This is evident in Supplementary Fig. 13a, where the backfolding occurs exactly where Ref. 5 asserts the CDW gap to lie. We conjecture that this backfolding is likely the gap that the ARPES is observing. Higher resolution ARPES data will likely be needed to detect the actual CDW gap.

We note that Seo *et al*.[6] have proposed from extended Hückel tight-binding calculations that the MMT arises from a CDW instability resulting from Fermi surface nesting. To explore this possibility further, we performed DFT calculations for $La_4Ni_3O_{10}$ using *Bmab* coordinates. We then fit the eight bands nearest to the Fermi energy with a Fourier series spline fit. The fit is shown in Supplementary Fig. 13a and the resulting Fermi surfaces in the k_z=0 plane in Supplementary Fig. 13b. Note the zone folding relative to a hypothetical undistorted *I*4/*mmm* structure. Since the bands 1 and 2 Fermi surfaces are hole-like, and the bands 3 and 4 Fermi surfaces are electron-like, nesting is a distinct possibility. To test this possibility, we first accurately determined the Fermi energy and then calculated the static susceptibility (χ) using a linear tetrahedron method. The resulting interband χ terms are shown in Supplementary Fig. 13c. Here we present a cut along (q,0,0). Almost equivalent results are found along (0,q,0), implying that the orientation of the density wave with **q** ∥ **b** is due to pinning by the structure. The band 2 to band 3 interband term shows a maximum at (0.5,0,0) but has subsidiary peaks at (0.5±0.11,0,0), with 0.61 lying close to the observed $1-q_s=0.62$.

Since we find considerable structure in the density of states near the Fermi energy, we recalculated χ by reducing the Fermi energy by 30 meV (Supplementary Fig. 13d). Now the side peaks become the global maxima, although they are shifted to (0.5±0.09,0,0). These results indicate that nesting is a distinct possibility. To improve on these results would require more sophisticated calculations that include not only spin matrix elements between the various band eigenvectors, but also potential correlation-induced energy shifts between the Ni 3*d* and O 2*p* states. For these reasons, we did not pursue further calculations along these lines, including the q_z dependence (which we find to be weak).

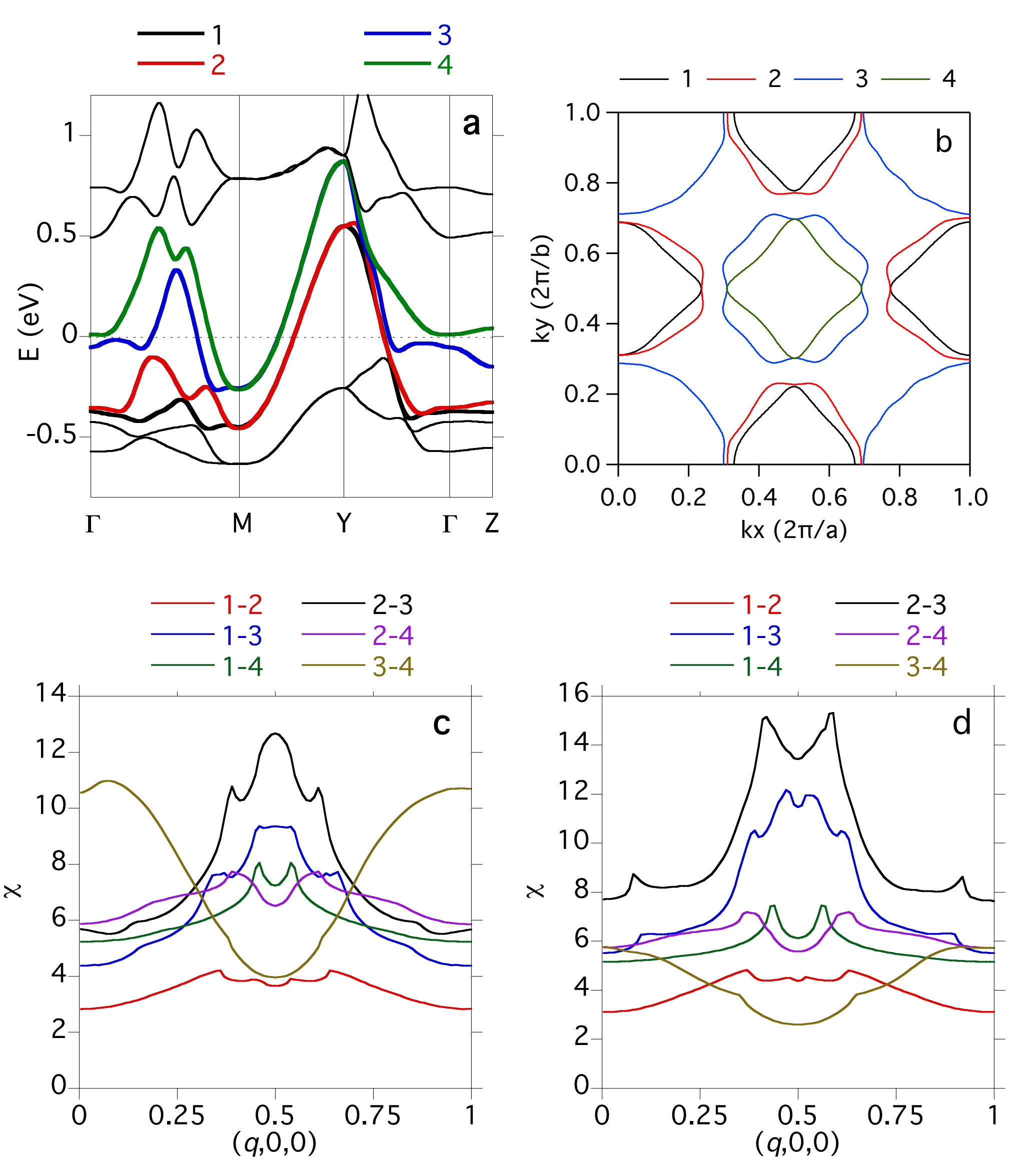

Supplementary Fig. 13 Band structure and Fermi surface nesting. **a**, Fourier series spline fit to the eight DFT bands nearest the Fermi energy (energies in eV with Fermi energy at 0). Here, *M*=(½,½,0), *Y*=(0,½,0), and *Z*=(0,0,1) in *Bmab* notation (2π/a, 2π/b, 2π/c units). The bands used in (b-d) are marked in bold and labeled. **b**, Fermi contours (k_z=0 plane) for the four energy bands crossing the Fermi energy, with bands 1,2 being hole-like and 3,4 being electron-like, implying the possibility of nesting. Decomposition of the susceptibility χ into various interband terms (ij, where i and j are band indices, with each being the sum ij+ji) at **c** the Fermi energy and **d** at 30 meV below the Fermi energy. Here, q is in 2π/a units. The outermost of the three peaks in **c** for the 2-3 interband term is at q=0.61, close to the observed SDW wavevector at 1-q_s=0.62. This peak becomes a global maximum in **d**, though it is shifted to q=0.59. This indicates that nesting is a potential cause of the SDW in $La_4Ni_3O_{10}$.

Supplementary References

1. Zhang, J. *et al.* High oxygen pressure floating zone growth and crystal structure of the layered nickelates $R_4Ni_3O_{10}$ (R=La, Pr). *Phys. Rev. Mater*. **4**, 083402 (2020).
2. Forgan, E. M. *et al.* The microscopic structure of charge density waves in underdoped $YBa_2Cu_3O_{6.54}$ revealed by x-ray diffraction. *Nat. Commun.* **6**, 10064 (2015).
3. Fawcett, E. Spin-density-wave antiferromagnetism in chromium. *Rev. Mod. Phys.* **60**, 209-283 (1988).
4. Lee, S., Chen, R. & Balents, L. Landau theory of charge and spin ordering in the nickelates. *Phys. Rev. Lett.* **106**, 016405 (2011).
5. Li, H. *et al.* Fermiology and electron dynamics of trilayer nickelate $La_4Ni_3O_{10}$. *Nat. Commun.* **8**, 704 (2017).
6. Seo, D. K., Liang, W., Whangbo, M. H., Zhang, Z. & Greenblatt, M. Electronic band structure and madelung potential study of the nickelates La_2NiO_4, $La_3Ni_2O_7$, and $La_4Ni_3O_{10}$. *lnorg. Chem.* **35**, 6396-6400 (1996).
